# Biases and complementarity in gut viromes obtained from bulk and virus-like particle-enriched metagenomic sequencing

**DOI:** 10.1128/spectrum.00013-25

**Published:** 2025-07-02

**Authors:** Yun Li, Chuqing Sun, Jiaying Zhu, Mingyan Geng, Min Li, Xing-Ming Zhao, Wei-Hua Chen

**Affiliations:** 1 Key Laboratory of Molecular Biophysics of the Ministry of Education, Hubei Key Laboratory of Bioinformatics and Molecular Imaging, Center for Artificial Intelligence Biology, Department of Bioinformatics and Systems Biology, College of Life Science and Technology, Huazhong University of Science and Technology 208338, Wuhan, Hubei, China; 2 Wallenberg Laboratory, Department of Molecular and Clinical Medicine and Sahlgrenska Center for Cardiovascular and Metabolic Research, University of Gothenburg 3570, Gothenburg, Sweden; 3 Institution of Medical Artificial Intelligence, Binzhou Medical University 272015, Yantai, China; 4 Department of Neurology, Zhongshan Hospital and Institute of Science and Technology for Brain-Inspired Intelligence, Fudan University 12478, Shanghai, China; 5 State Key Laboratory of Medical Neurobiology, Institutes of Brain Science, Fudan University 145751, Shanghai, China; 6 MOE Key Laboratory of Computational Neuroscience and Brain-Inspired Intelligence and MOE Frontiers Center for Brain Science, Fudan University 12478, Shanghai, China; 7 School of Biological Science, Jining Medical University 74496, Rizhao, China; Hôpital Saint-Louis, Paris, France

**Keywords:** virome, bulk-metagenomic sequencing, viral-like particle metagenomic sequencing, gut virus, gut phage, gut health

## Abstract

Due to varying sequencing strategies, current gut virome findings show significant variability. Specifically, bulk- and virus-like particle (VLP)-enriched metagenomic sequencing (termed bulk and VLP, respectively) present unique advantages and limitations, affecting viral genome discovery, taxonomic annotation, and community structure analysis. A comprehensive comparison of these strategies is crucial for thoroughly understanding the gut virome. This study comprehensively compared gut viromes identified from paired bulk and VLP data from 151 adult and 141 infant fecal samples. The VLP method showed superior performance to bulk in viral genome discovery in both data sets by recovering longer and more complete viral genomes, with higher sensitivity for low-abundant ones, resulting in a higher taxonomic annotation rate. However, we observed no correlations in the viral community structure (i.e., Shannon diversities) between bulk- and VLP-derived viromes, implying biases introduced during VLP enrichment. Such biases could be caused by the bacterial host features, such as the structural differences in cell walls and the prevalence and abundance of the viruses. Viruses that are of low prevalence, low abundance, or have Gram-positive bacteria as their hosts were enriched in VLP-derived viromes, in both the adult and infant data sets. Significant complementarity was observed between bulk and VLP viromes, with only about a quarter (26.7% in infants; 29.3% in adults) of VLP-viral genomes overlapping with bulk viruses. Together, our study identifies causal factors underlying the biases of bulk and VLP strategies in human gut virome studies and advocates the use of both strategies to enhance a comprehensive understanding of gut viromes.

**IMPORTANCE:**

The two mainstream gut phageome profiling strategies, namely bulk and virus-like particle (VLP), generated significantly overlapped results and have their own merits and drawbacks. Particularly, VLP exhibits higher efficiency in obtaining more, longer, and more complete viral genomes. However, VLP sequencing has the potential to alter the natural structure of viral communities, often resulting in the identification of viruses with lower prevalence and those specifically associated with Gram-positive bacterial hosts. While bulk metagenome features a more stable and diverse community, which can well reveal the interactions between viruses and bacteria. Nevertheless, bulk sequencing can suffer from lower coverage, leading to fragmented sequences and potentially missing some viral species. Therefore, it is essential to recognize that these methods are complementary rather than competitive in the comprehensive characterization of the gut phageome.

## INTRODUCTION

The human gut harbors trillions of microbes comprising bacteria, viruses, fungi, and other microorganisms, ordered by their relative numbers (1, 2). In recent years, the viral component of the gut, consisting mostly of bacteriophages and archaeal viruses (hereafter referred to as phages), has garnered significant attention due to its crucial role in modulating the gut micro-ecosystem and its links to various human diseases (3). In addition, gut phages show great promise for precision manipulation of the gut bacteriome and as potential antimicrobial agents, given their vast numbers and specificity in targeting bacterial hosts (2).

Currently, there are two main strategies to explore the human gut virome, including bulk-metagenome sequencing (hereafter referred to as bulk) and viral-like particle metagenome sequencing (hereafter referred to as VLP). The bulk approach sequences all the DNA in a gut sample, allowing for the simultaneous detection of both bacteria and viruses (4, 5). Due to the low overall total viral abundance (~5.8%) in the gut (2, 6), researchers often rely on viral recognition tools to identify viral genomes from assembled metagenomic data (7–9). Several large catalogs of human viral genomes have been created in this method, including the Gut Phage Database (GPD) (10), the Metagenomic Gut Virus catalog (MGV) (11), and the Cenote-Taker 2-compiled Human Virome Database (CHVD) (12). However, bulk suffers from a few limitations (13). First, due to the overall low viral abundance, bulk tends to result in low coverage of the gut virome (13–16). Second, bulk often fails to distinguish prophages (viral genomes integrated into bacterial chromosomes) from active replicating viruses due to their shared sequence identity with host bacterial DNA (17–19). As a result, there is a risk of misidentifying some inactive or incomplete prophages as phages, leading to the overestimation of virome complexity, viral diversity, misinterpretation of viral abundance, and limited understanding of viral activity (20). Last but not least, bulk is biased toward highly abundant viruses (21), resulting in the loss of certain low-abundant viruses.

The VLP sequencing approach involves VLP enrichment followed by metagenomic sequencing. Recently, several large-scale studies have constructed representative viral genome catalogs including the Global Virome Database (GVD) (22), Danish Enteric Virome Catalog (DEVoC) (23), and the Chinese Human Gut Virome (CHGV) (24). VLP can improve the detection sensitivity of low-abundant and rare viruses. However, it comes with inherent limitations. VLP requires stringent experimental standards and involves multiple steps, making it technically more demanding and time-consuming than bulk metagenomic sequencing (25). The need for a large sample volume poses a particular challenge, especially when working with limited sample resources. Furthermore, biases may be introduced during VLP, such as the potential loss of prophages integrated into bacterial genomes during filter-based enrichment or biases stemming from amplification preferences. These biases can result in an incomplete or skewed representation of the virome (26, 27). In addition, the yield of VLPs obtained from VLP enrichment can be relatively low, requiring further amplification to meet the sequencing requirements (28). Despite efforts to minimize contamination, there is still a risk of bacterial contamination in VLP-enriched samples, which can affect the accuracy of the downstream virome analysis (29). Finally, although experimental procedures could significantly enrich VLPs from fecal samples, contamination could happen and account for significant proportions of the VLP sequencing reads (30). Consequently, researchers also rely on viral recognition tools to identify viral genomes, similar to that of the bulk data process (7–9). These limitations highlight the importance of carefully considering the trade-offs and potential pitfalls when choosing VLP as an approach for studying gut viromes.

Despite the successful application of both methods and the resulting massive expansion of identified gut viral genomes, as mentioned above, the systematic comparison between the two methods has not been extensively studied. Previous studies have shown that bulk and VLP viromes exhibit different compositions, but the data sets used contained mostly unpaired (different samples were sequenced with bulk and VLP) or only a few paired (samples are both sequenced with bulk and VLP) sequencing data. For example, Gregory AC et al. (22) constructed a database of gut viruses using ten unpaired bulk- and VLP-fecal samples and revealed 8.5% shared phage genomes between the two strategies. This observation was later supported by a comprehensive multi-environment study showing systematic differences between bulk and VLP methods across ecosystems, though with limited representation of human gut samples (*n* = 10) (31). Wang G, et al. (13) examined paired data from five fecal samples and found that, on average, only 16.4% of the phages identified by VLP were shared with those from bulk. They also noted differences in the distribution of phage families and higher within-sample diversity in bulk than in VLP. A subsequent human fecal virome-focused comparison further validated these phage detection biases and reinforced the higher reproducibility of bulk sequencing, albeit in a small cohort (*n* = 10) (32). Zhang F, et al. (33) reported that the two methods were equally effective in phage detection, with no difference in the number of observed species between them using 12 fecal samples. In addition, Zeng et al. (34) utilized the largest comparison so far using 141 pairs of infant samples (<1 year old) and found the different efficiency of detecting phages and the correlation of bacteriome and virome in bulk and VLP. However, a comprehensive evaluation using a large number of samples in adults, for whom most gut virome studies focused on, is still needed to investigate and understand the extent of agreement between these two methods in terms of viral community characteristics. This will contribute to a more comprehensive understanding of the gut virome, ensuring the reliability and comparability of future studies.

In this study, we aim to comprehensively and comparatively analyze the viral genomes obtained by the two methods. To achieve this, we systematically analyzed paired samples (i.e., those that were subjected to both bulk and VLP sequencing) from 151 healthy adults (24) and compared them with the results using the paired samples of previously published 141 healthy infants (35). Our analysis using the two datasets reveals notable differences in bulk- and VLP-derived gut viromes in various aspects, including community characteristics, genomic features, taxonomy annotation, and unique attributes of each method. Among which, VLP exhibits higher efficiency in obtaining more, longer, and more complete viral genomes with a higher taxonomy annotation rate. However, VLP alters the structures of the actual viral communities and tends to yield viruses with lower prevalence and those whose hosts are Gram-positive bacteria. On the other hand, bulk features a more stable and enriched community, which can well reveal the interactions between viruses and bacteria. Furthermore, we found significant complementarity between the viruses obtained by bulk and VLP. Together, our results underscore the importance of combining both bulk and VLP in virome studies to obtain a comprehensive understanding of the virome community.

## RESULTS

### VLP outperforms bulk metagenomic sequencing by recovering more, longer, and complete gut viral genomes

**Fig 1:**
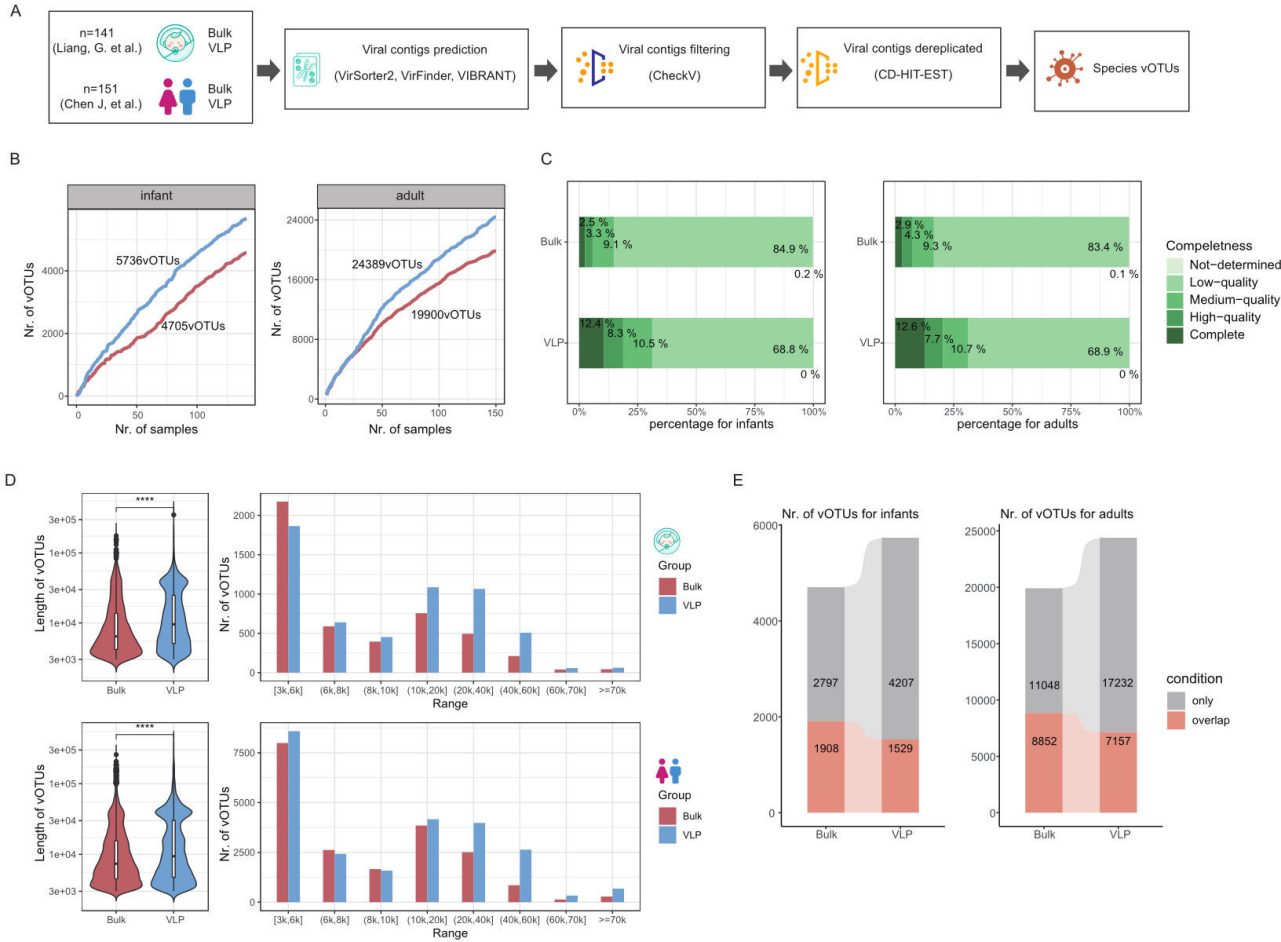
Profiling of the human gut virome by bulk-metagenome sequencing (bulk) and viral-like particle-metagenome sequence (VLP). (**A**), Overall experimental design and process of the analysis. (**B**), Accumulation curve of vOTUs with sample number derived from the two methods. The right panel is infant data sets, and the left panel is adult data sets. (**C**) The distribution of different levels of completeness was determined by CheckV. The right panel is infant data sets and the left panel is adult data sets. (**D**) The left panel shows the comparison of the length in viral genomes by bulk and VLP. The right panel shows the length distribution of viral genomes divided into equal-length bins for both the bulk and VLP methods. The right panel is infant data sets, and the left panel is adult data sets. Color refers to groups: bulk (red) and VLP (blue). (**E**) The similarity of vOTUs of bulk and VLP by ANI > 95% (left for infants, and right for adults). Colors refer to groups: only (gray) and overlap (red).

To systematically characterize and compare the gut viromes obtained from bulk-metagenome sequencing (bulk) and viral-like particle metagenome sequencing (VLP), the two mainstream methods used by researchers, we analyzed 584 fecal metagenome samples, including 151 healthy adults and 141 infants that were subjected to both bulk and VLP sequencing (24, 35) and went through identical bioinformatic pipeline for downstream analysis (Fig. 1A, Materials and Methods). Briefly, after the removal of the human host, the remaining sequencing reads were assembled using MEGAHIT (36), retaining contigs with >3 kb in length. Viral contigs were identified using three widely recognized viral detection tools, VirSorter2 (9), VirFinder (8), and VIBRANT (7) (see “Materials and Methods”). The obtained viral sequence per sample is combined by group (*n* = 19,032 in bulk and *n* = 12,857 in VLP of infants; *n* = 86,701 in bulk and 122,756 in VLP of adults). The combined viral sequence was first quality-filtered using CheckV (37). The filtered sequences were then dereplicated based on an average nucleotide identity (ANI) threshold of 95% global sequence identity and alignment fraction of 85% using CD-HIT (38) (Materials and Methods). In total, we obtained 4,705 and 5,736 non-redundant species-level viral operational taxonomic units (vOTUs) in bulk and VLP data of infants and 19,900 and 24,389 vOTUs in bulk and VLP data of adults, respectively. We found the cumulative number of vOTUs per sample, and VLP is consistently higher than bulk (Fig. 1B).

Next, we compared the qualities of the vOTUs obtained from the two methods and found that VLP consistently generated more and higher-quality phage genomes. Particularly, after stratifying the vOTUs into five quality groups according to their CheckV (37) scores, we found that VLP generated higher proportions of high-quality and complete vOTUs (with >90% genomic completeness) than the bulk method in both infant and adult data sets (Fig. 1C). In addition, VLP-vOTUs were consistently longer than the bulk-vOTUs (with medians of 9,569 vs. 6,423 in infants, and 9,430 vs. 7,368 adults for VLP and bulk, respectively; Wilcoxon rank-sum test, *P* < 0.0001; Fig. 1D), and a higher proportion of vOTUs longer than 10kbp in VLP than bulk (48.4% vs. 32.9% in infants and 48.3% vs. 38.3% in adults for VLP vs. bulk, respectively; Fig. 1D; Table S1).

We also compared the overlaps of the vOTUs obtained by VLP and bulk. Using ANI of 95% and aligned fraction (AF) of 85% as the threshold as previously reported (22), we found that 40.6% (1,908 out of 4,705) of the bulk-vOTUs overlapped with 26.7% (1,529 out of 5,736) of the VLP-vOTUs in infants, and 44.5% (8,852 out of 19,900) of bulk-vOTUs overlapped with 29.3% (7,157 out of 24,389) of the VLP-vOTUs in adults (Fig. 1E; Table S1). These results suggest that the shorter bulk vOTUs are actually fragments of the longer genomes obtained by VLP.

Together, VLP consistently overperformed bulk by obtaining longer and more complete gut viral genomes. However, VLP and bulk exhibited significant complementarity in gut viral genome detection.

### VLP significantly alters viral community structure as compared to bulk

To characterize the community characteristics of the vOTUs from VLP and bulk, we determined their relative abundances by first aligning the clean reads to the vOTUs using BWA (39) and then calculating the reads per kilobase million (RPKM) values in each of the samples (Materials and Methods). We then determined the “presence” of a vOTU in a sample if over 75% of its length was covered by the aligned reads from that sample (40), and then calculated the relative abundances of all vOTUs that were presented in a sample as their percentages of the RPKM values out of total in the sample.

**Fig 2:**
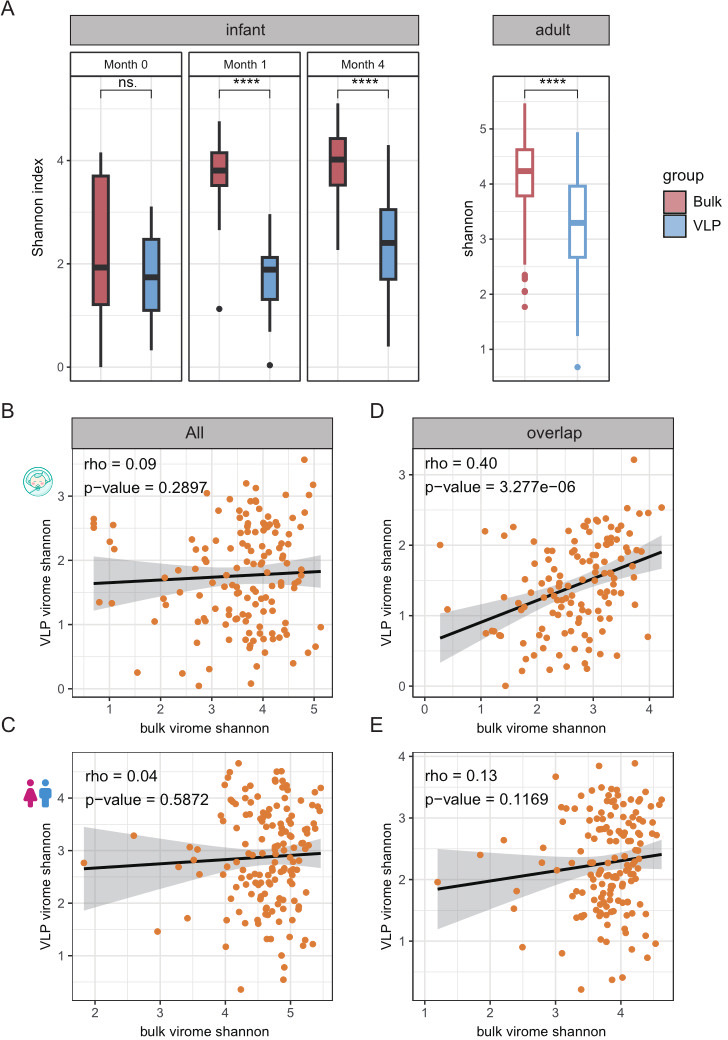
Distinct community characteristics of the human gut virome by bulk-metagenome sequencing (bulk) and viral-like particle-metagenome sequence (VLP). (**A**), Comparison of within-sample Shannon diversity of vOTUs (left for infants, and right for adults). Paired Wilcoxon rank-sum test: ns.: *P* > 0.05; *P < 0.05; **P < 0.01, ***P < 0.001, ****P < 0.0001. Colors refer to groups: bulk (red) and VLP (blue). (**B and C**), Spearman’s rank correlation of alpha diversity (Shannon index, all of the virome on the left and the overlap of the virome on the right) between bulk-virome and VLP-virome with two sides. B refers to infants, and C refers to adults.

We first compared the community diversity characteristics between the bulk- and VLP-derived gut viromes. We found that the richness (i.e., the number of vOTUs in each sample) of infants in bulk is higher than VLP (median 89 in bulk; median 57 in VLP), whereas there was no difference in adults (*P* = 0.37; median 436.5 in bulk; median 417 in VLP; paired Wilcoxon rank-sum test) (Fig. S1A). Thus, the higher total number of vOTUs in VLP was due to fewer between-sample overlaps than the bulk, as evidenced by the accumulation curve shown in Fig. 1B. This is also supported by the significantly higher between-sample dissimilarities in VLP samples than in bulk samples (paired Wilcoxon rank-sum test, *P* < 2.2e-16; Fig. S1B). In addition, we observed a higher Shannon index in the bulk samples (paired Wilcoxon rank-sum test, *P* < 2.2e-16), likely driven by higher evenness (paired Wilcoxon rank-sum test, *P* < 0.05) than the VLP samples (Fig. 2A; Fig. S1C), suggesting a more balanced abundance distribution of vOTUs in bulk.

More importantly, we found no significant correlation in the Shannon diversity between bulk- and VLP-derived viromes (rho = 0.09, *P* = 0.2897 in infants; rho = 0.04, *P* = 0.5872 in adults) (Fig. 2B and C). When limiting our analysis to vOTUs identified by both bulk and VLP methods (overlapped vOTUs), we found increased correlations in the Shannon diversity between bulk- and VLP-derived viromes (rho = 0.40, *P* = 3.227e-06 in infants; rho = 0.13, *P* = 0.1169 in adults) (Fig. 2D through E); however, the correlation remained statistically insignificant in adults. These results collectively implied that the community structure of the gut virome was altered (i.e., both community members and vOTUs abundances) by VLP, likely due to experimental procedures applied to extract the virus-like particles.

To address potential concerns regarding genome fragmentation biases in bulk sequencing, we performed additional quality control using CheckV completeness filtering (≥50% and ≥90% thresholds). Notably, after controlling for genome completeness, VLP samples showed significantly higher viral richness than bulk (*P* < 0.0001), indicating initial bulk richness estimates were inflated by fragmented genomes. The higher Shannon diversity in bulk samples remained robust when CheckV ≥50%, confirming its advantage in capturing balanced communities. VLP maintained altered community structures at all completeness levels (Fig. S1D through I). In conclusion, the greater heterogeneity in VLP-derived communities persisted even after quality filtering, supporting our hypothesis that VLP enrichment alters detectable community structure.

We then explored other factors that could contribute to the above observations, especially the lifestyles of gut viruses. Strikingly, we observed marked differences between lytic and temperate phages: temperate phages showed significantly higher richness than temperate phages in both bulk and VLP samples (*P* < 0.0001, Wilcoxon test), whereas lytic phages exhibited higher Shannon diversity (*P* < 0.01) (Fig. S2A through D). This pattern held true across both infants and adults, suggesting fundamental ecological differences between these two phage types. However, we did not find significant correlations between bulk- and VLP-derived virome for either of the temperate or lytic phage types (Fig. S2E and F), suggesting that the lack of correlation predominantly stems from differences between bulk and VLP samples.

We also evaluated the correlations in community structures (i.e., Shannon diversity) between the gut virome and bacteriome, because as obligate parasites of bacteria, phages are known to significantly impact the structure of the bacteriome in the human gut through bacterial lysis and integration as prophages (2, 34, 41, 42). We calculated the taxonomic profiles of the gut bacteriome from the bulk data using MetaPhlAn4 (ver 4.0) (43). We observed significant positive correlations between the bulk virome and bacteriome Shannon indexes (Spearman Correlation’s rho = 0.65, *P*-value < 2.2e-16 in adults, and rho = 0.48, *P*-value = 3.651e-09 in infants) (Fig. S3A and B), consistent with a previous study (5, 34). However, no correlation was observed between the VLP virome and bacteriome Shannon (rho = −0.02, *P*-value = 0.7917 in adults, and rho = 0.02, *P*-value = 0.3899 in infants) (Fig. S3A and B). We speculate it is due to the biased VLP abundance as mentioned above, which, in turn, led to a bias in the diversity of the viral community.

Collectively, our findings suggest that VLP significantly altered the viral community structure as compared to bulk.

### High-abundance and prevalent viruses are more likely to be recovered by both methods

We next explored why some viral genomes could be detected by both methods, while others were method-specific. We categorized the bulk-vOTUs into two groups, namely “bulk-only”and “bulk-overlap,” according to whether they shared sequence similarity (i.e., >95% ANI and >85% AF) with the VLP-vOTUs, and the latter into “VLP-only” and “VLP-overlap” accordingly.

**Fig 3:**
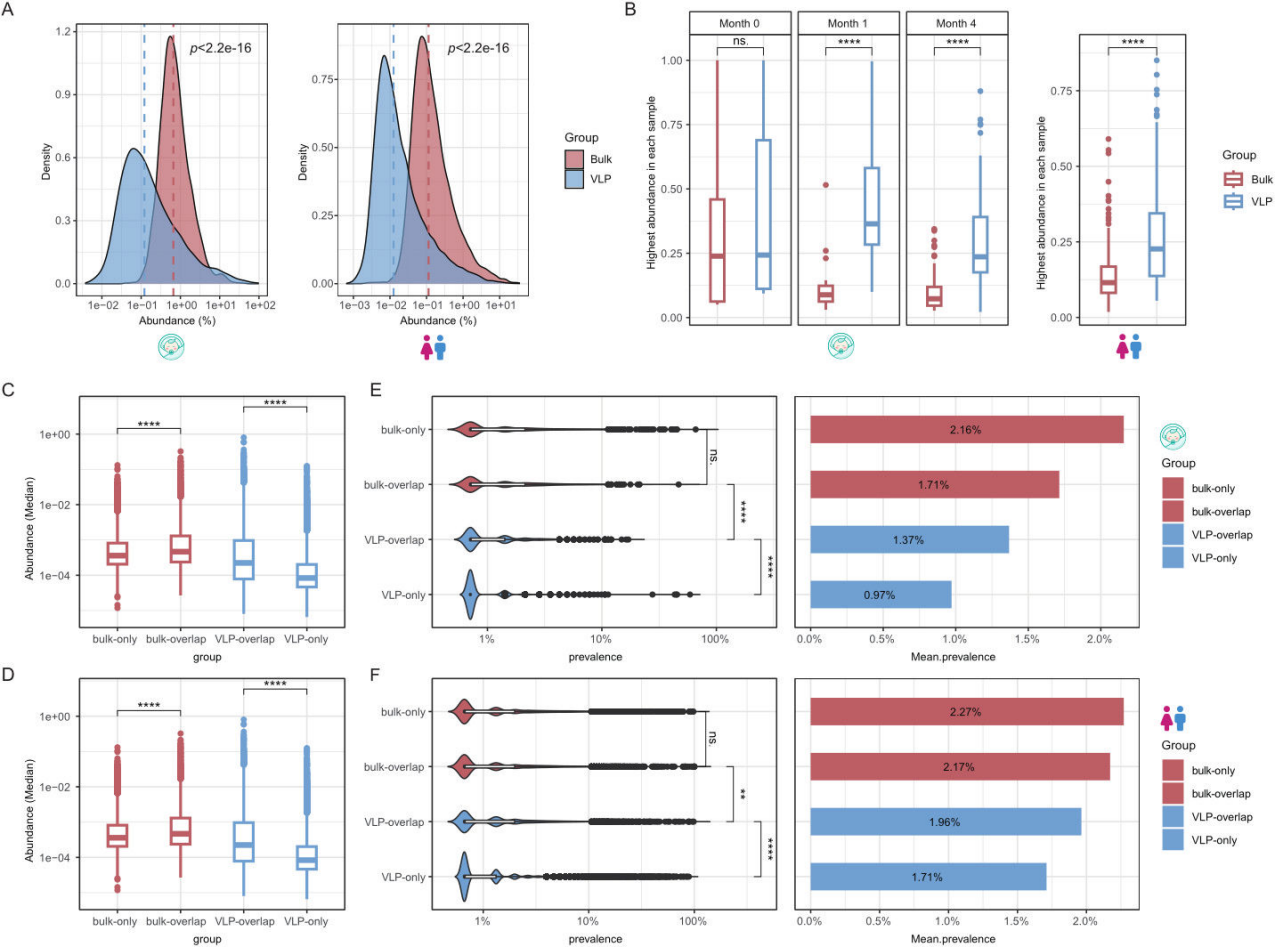
The distribution of abundance and prevalence of the human gut virome by bulk-metagenome sequencing (bulk) and viral-like particle-metagenome sequence (VLP). (**A**), The distribution of the relative abundance of vOTUs (all) (left for infants, and right for adults). (**B**), The relative abundance of the most abundant vOTUs in each sample (paired Wilcoxon rank-sum test, left for infants, and right for adults). (**C and D**) The comparison of the relative abundance of the vOTUs of bulk-only, bulk-overlap, VLP-overlap, and VLP-only (“only” refers to the vOTUs that only appeared in bulk or VLP; “overlap” refers to the vOTUs that appeared in both bulk and VLP). (**E and F**) The comparison of the prevalence of the vOTUs of bulk-only, bulk-overlap, VLP-overlap, and VLP-only. Wilcoxon rank-sum test: ns.: *P* > 0.05; *P < 0.05; **P < 0.01, ***P < 0.001, ****P < 0.0001. Colors refer to groups: bulk (red) and VLP (blue).

We observed that the overall abundance of VLP-derived vOTUs (RPKM; see Materials and Methods) was significantly lower than that of bulk-derived vOTUs in both infant and adult data sets (*P* < 2.2e-16, Wilcoxon rank-sum test; Fig. 3A; Fig. S4A), likely due to the greater number of viral genomes recovered by the VLP method. Nonetheless, the most abundant vOTUs per sample exhibited higher abundance in VLP libraries (Fig. 3B), indicating that despite broader recovery, the VLP approach remains biased toward highly abundant viruses, potentially due to extraction-related factors.

We observed that the overlapped vOTUs of the bulk and VLP groups had significantly higher abundances than their respective “only” groups (*P* < 2.2e-16, Wilcoxon rank-sum test; Fig. 3C and D), indicating that high abundance is a key factor for a virus to be captured by both methods. As expected, the overlapped vOTUs in the VLP samples have a higher prevalence than the “only” group. In bulk samples, no significant difference in prevalence was observed between “only” bulk-vOTUs and overlapped-vOTUs (*P* = 0.07 in infants, *P* = 0.83 in adults, Wilcoxon rank-sum test; Fig. 3E and F). The trend of prevalence (mean prevalence gradually declines by “bulk-only,” “bulk-overlap,” “VLP-overlap,” and “VLP-only”) shows that the bulk method could detect high-prevalent viruses better than VLP. However, caution should be taken because, due to frequent viral-host (mostly phage-bacteria) gene exchanges, the viral prevalence could be overestimated because of the bacterial reads in the bulk.

Notably, when examining phage lifestyles across all detected vOTUs, we found temperate phages exhibited significantly higher median abundance than lytic phages in both bulk (1.1-fold higher, *P* < 0.01) and VLP samples (1.4-fold higher, *P* < 0.0001; Fig. S4A). Similarly, temperate phages showed greater prevalence than lytic phages (*P* < 0.01; Fig. S4B). These patterns were consistent across both infant and adult data sets. The consistency of these findings demonstrates that the niche differentiation between lytic and temperate phages is inherent and remains unaffected by the choice of sequencing methodologies.

### Bacterial host characteristics as key determinants for taxonomical biases caused by VLP

**Fig 4:**
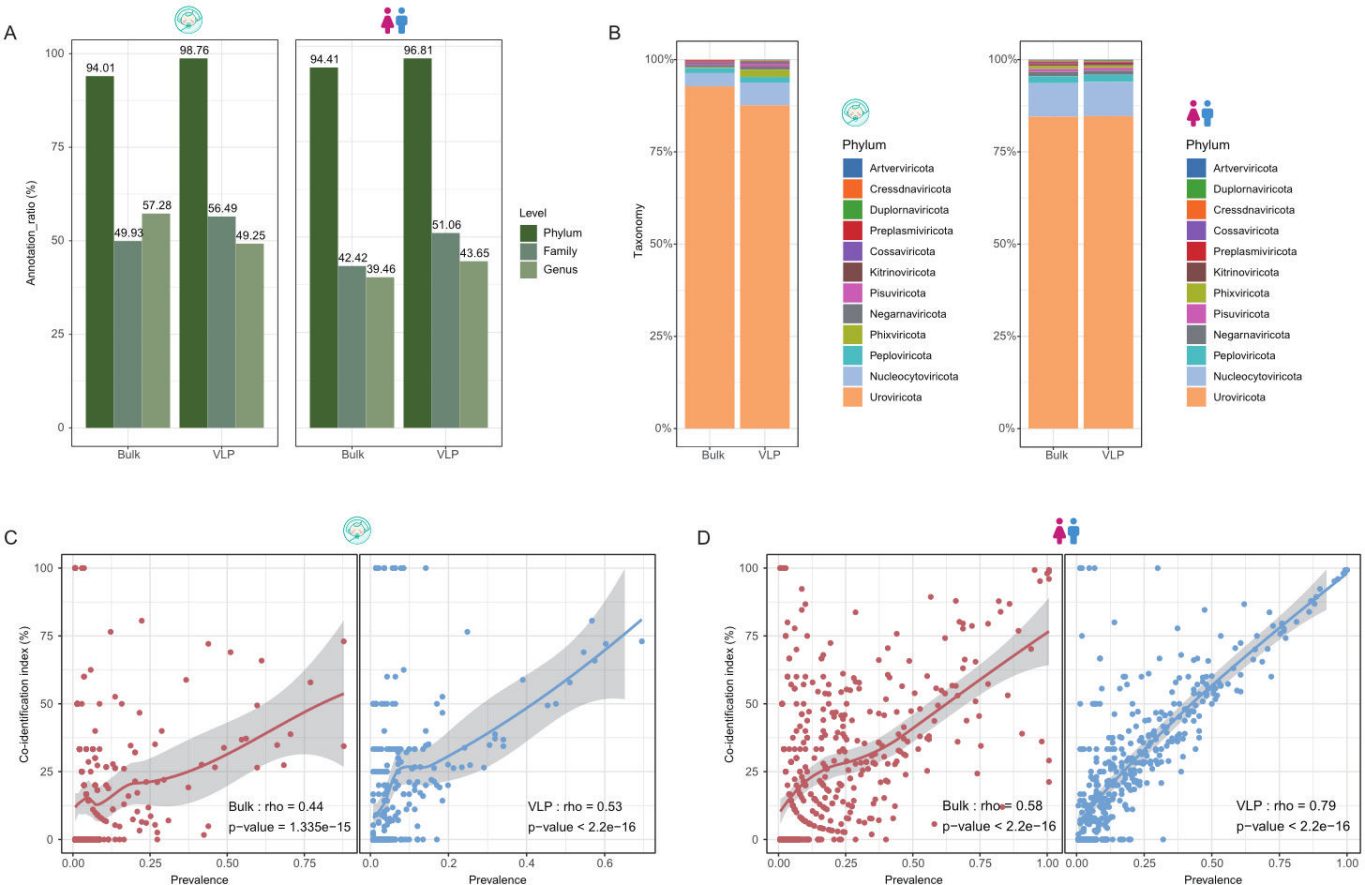
The consistency of taxonomy annotation for the human gut virome by bulk-metagenome sequencing (bulk) and viral-like particle-metagenome sequence (VLP). (**A**) The annotation rate in different levels of the vOTUs by bulk and VLP (left for infants, and right for adults). (**B**) Viral composition of the vOTUs by bulk and VLP at the phylum level (left for infants, and right for adults). (**C and D**) The correlation of the consistent rate of the two methods and prevalence in the genus level (the left panel is bulk and the right panel is VLP; C is infants and D is adults).

Using our taxonomic annotation pipeline (Materials and Methods), we were able to assign over 90% of the vOTUs to known phyla, but only 40%–60% of them to known families and genera (Fig. 4A). Generally, the VLP-vOTUs exhibited higher annotation rates than the bulk at the three taxonomic levels (Fig. 4A), likely due to longer and more complete genomes obtained from VLP (Fig. 1C and D).

We further explore taxonomical biases introduced by VLP as compared with the bulk. At the phylum levels, the species distributions in the bulk and VLP are essentially the same in the adult data set, but in the infant data set, *Uroviricota* was much less represented in the VLP, while *Nucleocytoviricota* and *Phixviricota* are significantly more represented (Fig. 4B). At the genus level, we observed distinct viral genera in bulk- and VLP-vOTUs (Fig. S5C and D).

To quantify the effects of the viral prevalence of a taxonomic clade on its likelihood of coincidental identification (co-identity) by both methods in the same sample, we calculated the percentage of paired samples where both bulk and VLP could simultaneously annotate the same viral genus, relative to all samples that could annotate this genus. As shown in Fig. 4C and D, we found significant positive correlations between the genus prevalence and the likelihood of co-identification, in both bulk and VLP samples (*P* < 1.3e-15, rho = 0.44 ~ .79; Spearman’s rank correlation test); the trends were stronger in VLP samples (i.e., from which the genus prevalence was calculated) than the bulk, and also stronger in adults than in infants. These results indicate that highly prevalent viruses are more likely to be identified by both methods.

**Fig 5:**
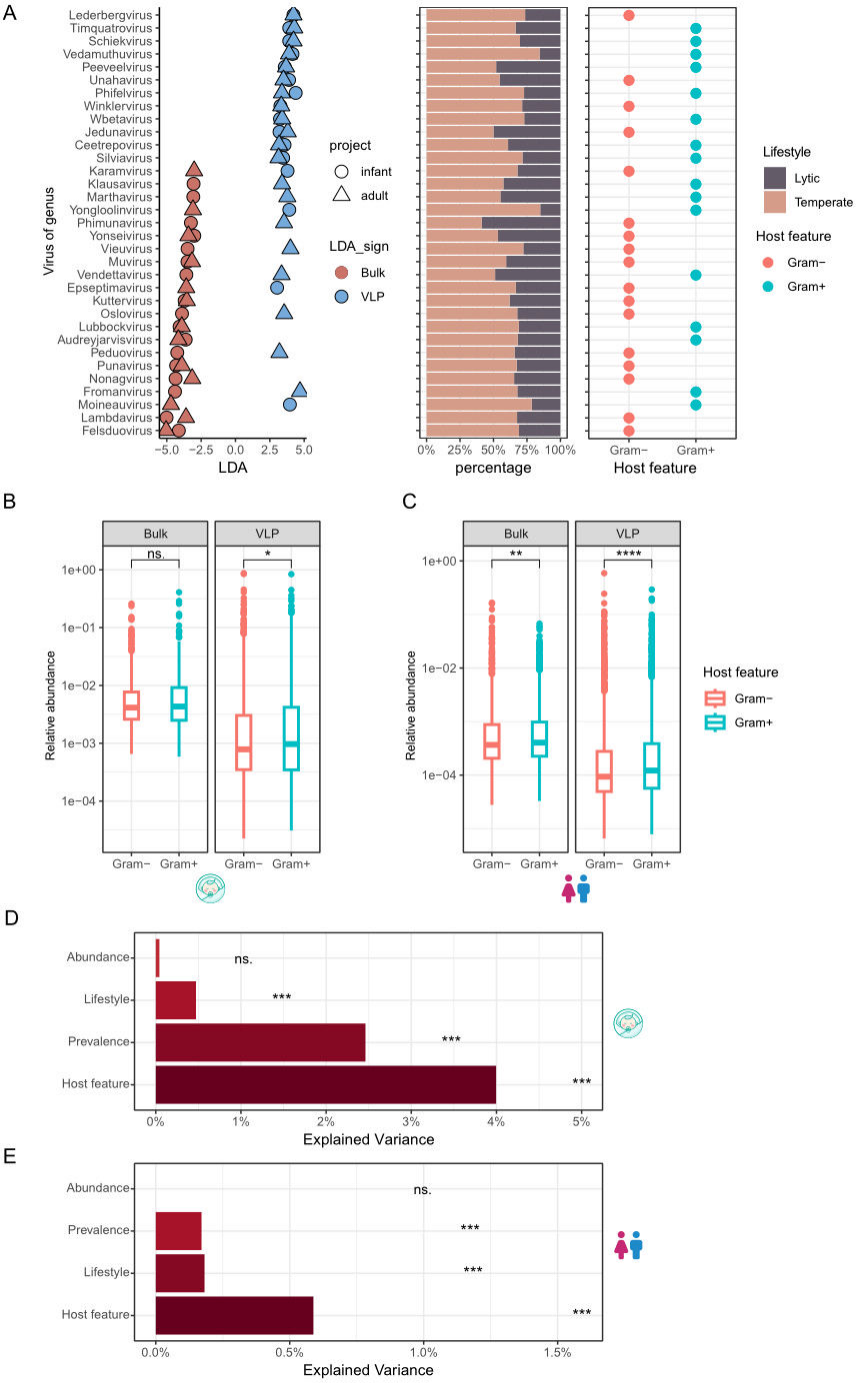
Enrichment and characterization of phages in bulk-metagenome sequencing (bulk) and viral-like particle-metagenome sequence (VLP) from infant and adult samples. (**A**) Enrichment of genera in bulk and VLP samples from infants and adults: The left panel shows that 11 genera are consistent between infant and adult data sets, while the remaining genera are not consistent. The middle panel illustrates the lifestyle characteristics of these genera, and the right panel depicts the host features associated with these genera. (**B and C**) Comparison of the abundance of phages targeting Gram-positive vs. Gram-negative bacteria in bulk and VLP samples. The left panel is infants, and the right panel is adults. (**D and E**) Effect size (i.e., explained variance or R^2^) explained by median abundance, prevalence, lifestyle, and host features as determined by PERMANOVA, using Bray-Curtis distances between all viral community profiles within each method group (VLP vs bulk) to assess the contribution (top for infants, and bottom for adults). FDR < 0.001 (***) refers to the significance of each factor, and FDR > 0.05 (ns.) refers to no relevance.

To quantify the genus-level biases, we used LEfSe (LDA EFfect Size) to identify differentially abundant genera between bulk and VLP. A total of 65 genera were identified at a threshold of |LDA| > 3.0 (linear discriminant analysis) and *P* < 0.05 (Table S2) in infants, including 27 VLP-enriched and 38 VLP-depleted; a total of 98 genera were obtained in adults, including 52 VLP-enriched and 46 VLP-depleted. Particularly, 11 genera showed consistent enrichment by VLP in both infant and adult data sets, while only nine showed consistent depletion (Fig. 5A; Fig. S6 and S7). We did not find significant trends in the lifestyle distributions (i.e., the proportion of temperate and lytic phages) among the differentially abundant genera, indicating that VLP is not biased toward lytic phages as we would expect. However, we did observe a trend that the VLP-enriched genera were more often associated with Gram-positive bacteria (Fig. 5A). For example, the hosts of most of these consistently enriched phages were Gram-positive bacteria *Enterococcus*, *Staphylococcus,* and *Bacillus* (Fig. S8). These results were likely since Gram-positive bacteria usually release more phage particles because of their relatively simple cell wall structure (44). By contrast, the process of releasing phage particles by Gram-negative bacteria may be hindered by the extracellular membrane, so the phages infecting Gram-positive bacteria tend to be more enriched and detected in particle enrichment experiments (44, 45). In fact, we found that phages associated with Gram-positive bacteria were generally more abundant than the Gram-negative associated ones, especially in VLP samples (Fig. 5B), further supporting our analysis.

To further excavate the critical factors that cause bias in the VLP, we performed the PERMANOVA analyses based on Bray-Curtis distances to estimate the effect size and probability value of abundance, prevalence, lifestyle, and host feature (i.e., the hosts of the phages are Gram-positive or Gram-negative bacteria), respectively (FDR < 0.05, 9999 permutations) on the gut virome profiled by VLP and bulk. The results showed that the host feature is the highest influence that affects the bias in the VLP (4.0% in infants and 0.6% in adults), followed by prevalence and lifestyle (Fig. 5C).

## DISCUSSION

With the increasing study of the human gut virome, different research groups using different sequencing protocols have led to general variation in results (30). There are commonly two next-generation sequencing research approaches when studying the gut virome: bulk-metagenome sequencing (bulk) and viral-like particle-metagenome sequencing (VLP), while both of them have advantages and disadvantages in comprehensively obtaining the gut virome (16). For example, there is a deletion of low-abundance virus in bulk (16) and a preference for VLP enrichment (30, 46). To address these discrepancies, researchers have attempted to compare the two methods, although most studies have relied on unpaired or minimally paired data sets. In this study, we utilized paired samples to thoroughly evaluate the distinctions and consistencies between the two methods. Furthermore, our sample size (*n* = 151 adults and *n* = 141 infants) significantly exceeds that of prior studies (13, 22), enabling more comprehensive comparisons and generalizable conclusions.

Our findings indicate that VLP sequencing is more effective in capturing vOTUs. In addition, the vOTUs in VLP are longer and more complete. Of course, the choice of assembly and identification software used can also affect the results (47, 48). By comparing the average nucleotide similarity of genomes obtained through both methods, we identified consistencies and discrepancies. In the infant data sets, 26.7% of the viral contigs identified by VLP sequencing are shared with 40.6% of those identified by bulk sequencing. In the adult data sets, 29.3% of VLP-identified contigs are shared with 44.5% of bulk-identified contigs. This suggests that bulk sequencing tends to capture shorter, fragmented viral contigs, potentially due to viruses accounting for only 5.8% of bulk sequencing content (2, 6, 49). Furthermore, there are significant differences between the viral genomes obtained by the two methods, covering over 40% of the bulk contigs, which is consistent with previous research findings (13, 22, 34).

Several studies have highlighted discrepancies in viral communities derived from bulk and VLP samples (13, 33), often pointing to a higher Shannon index in bulk samples without delving into the underlying causes (13). Our study suggests that the uneven abundance distribution of vOTUs in VLP samples may explain this phenomenon. Many vOTUs in VLP samples exhibited biased abundance, predominantly concentrated in the “only” category between the two methods. This distribution appears unrelated to the virus lifestyle. Moreover, while richness may appear greater in bulk samples (33), this difference primarily stems from the variability between VLP samples. In addition, our calculations were based on relative abundance, acknowledging that RPKM-based relative abundance can be significantly affected by the number of viral reads (50), although this did not seem to impact the infant data set. Furthermore, since we did not directly compare the abundance of vOTUs obtained by the two methods within the samples, our final results remain unaffected.

The interaction between bacteria and bacteriophages has been extensively investigated in previous studies. Recently, Zeng et al. (34) reported a robust correlation in community richness between the bulk-phageome and the bacteriome (*P* < 2.2e-16, cor = 0.56), which is consistent with the intricate co-evolution and mutual dependence observed within gut microbiota (5, 51). They also noted a weaker yet significant correlation in richness between the VLP-phageome and the gut bacteriome (*P* = 0.005, cor = 0.04). By contrast, our study of 151 paired adult samples revealed no such correlation between the VLP-phageome and the gut bacteriome (*P* = 0.0735, cor = 0.15) (Supplementary Figure S9). We postulate that biases introduced during VLP extraction may account for this discrepancy. Our findings are supported by recent observations in wastewater samples by Zhang J, et al. (52), despite the comparative ease of phage extraction from aquatic environments. Nevertheless, the simplified VLP extraction process introduces notable biases.

Our results demonstrate that viral genomes obtained through VLP exhibit higher annotation rates compared to those from bulk samples, likely due to the more complete viral genomes obtained from VLP. In addition, we identified a higher proportion of eukaryotic viruses in VLP (Supplementary Figure S10). Previous studies have compared viral species obtained through these two methods, but limitations such as small sample sizes and unmatched VLP and bulk samples have hindered a clear understanding of species distribution differences (13, 34). By leveraging a larger data set, we explored inter-group differences at the genus level, finding that found that it was predominantly phages of Gram-positive bacteria that were enriched in VLP. While some phages were consistently enriched across both methods in infant and adult data sets, 11 phages exhibited opposite enrichment directions, which we speculate may be age-related (34).

The identification of unique viromes by each method suggests that both VLP and bulk are capturing distinct aspects of the viral community. Excluding possible errors and biases, both methods may reveal valuable information that the other method did not detect. Further exploration of these unique species and genera, along with their ecological and functional features, may provide new insights into the diversity and dynamics of viral communities. The use of multiple methods can provide a more complete understanding of the functional potential and metabolic capabilities of microbial communities. By combining multiple data sets, we can overcome the limitations of individual methods and better understand the complex relationships between microbiome composition and function.

The observed methodological biases must be interpreted relationally that VLP’s biases are defined relative to bulk, and vice versa. For instance, VLP’s apparent underrepresentation of certain phage groups may actually reflect bulk’s overrepresentation of integrated prophages. Similarly, bulk’s higher Shannon diversity could indicate a technical artifact (from genome fragmentation). This interdependence highlights the need for method-aware interpretation of virome data. Our analysis focused on the two largest available gut virome data sets and excluded studies that contained few paired samples (<20 human samples) (13, 22, 31–33). However, we do feel that future studies incorporating emerging cohorts could further validate the findings presented in this study.

In summary, VLP and bulk are complementary techniques, and combining both approaches can provide a more comprehensive and detailed analysis of the gut virome. This could deepen our understanding of the complex interactions between viruses and the gut microbiota, revealing their role in gut balance, disease development, and potential therapeutic interventions. Further research and technological advances are needed to overcome the challenges and biases posed by each method, ultimately achieving a more comprehensive and accurate description of the gut virome.

## MATERIALS AND METHODS

### Data source

The public sequencing data used in this study are available in the CNCB GSA database under the accession of PRJCA007087 and the NCBI SRA database under the accession of PRJNA835720 for the adults (*n* = 151) (24), and in the NCBI SRA database under the accession of PRJNA524703 for the infants (*n* = 141) (35). The data were obtained with paired bulk-metagenomic sequencing (bulk) and viral-like particle metagenomic sequencing (VLP).

### Raw data processing and genome assembly

Both bulk and VLP reads were processed with Trimmomatic v0.38 (53) (with parameter LEADING:3 TRAILING:3 SLIDINGWINDOW:15:30 MINLEN:50) to remove adaptors and trim low-quality bases; reads of 50 bp or less after trimming were discarded. Next, host contamination reads were removed by querying the reads against the human reference genome (hg38; GCA_000001405.15) using Bowtie2 (54) v2.4.2. After processing the bulk reads, bacterial annotation was performed on these clean reads by MetaPhlAn4 (43). Subsequently, the quality-controlled reads of bulk and VLP were assembled with MegaHIT v1.1.3 (36) (default parameters except option “-min-contig-len 1000”).

### Viral identification and acquisition of non-redundant vOTUs

The main method for viral gene identification is consistent with Zeng S, et al. (34). After assembly, all contigs > 3 kb in length in each sample were submitted to VirSorter v2.2.3 (9), VirFinder v1.1 (8), and VIBRANT v1.2.1 (7) for identification of viral populations. For VirFinder, the viral sequences with a result of score >0.9 and *P* < 0.01 were left. For VirSorter, we run with default settings (default: dsDNAphage, ssDNA, --min-score 0.5) with option “--keep-original-seq.” For VIBRANT, with the default setting except “-f nucl,” and for VLP with the additional option “-virome.” Following this, viral sequences identified from three distinct viral identifiers were aggregated for each sample, generating viral sequences in four groups: for infant data sets, 19,032 in bulk samples, and 12,857 in VLP samples; for adult data sets, 86,701 in bulk samples, and 122,756 in VLP samples. Thereafter, the viral populations were filtered by CheckV v1.0.1 (37) (a, longer than 3 kb; b, the number of viral genes > the number of host genes; c, kmer_freq ≤ 1; and d, without warning “>1 viral region detected” and “contig >1.5 × longer than expected genome length”), and further dereplication by CD-HIT v4.6.8 (38) (parameters: -c 0.99 g 1 M 0 -d 0 n 10) to obtain viral sequences based on the two methods. The nucleotide BLAST database of both viral sequences was built by makeblastdb (option “-dbtype nucl”) from blast v2.13.0, and the pairwise comparisons were generated by blasting all viral sequences all-against-all with blastn (option “-max_target_seqs 10,000”). Finally, clustering of viral sequences into species-level vOTUs was carried out using two custom scripts (anicalc.py and aniclust.py) from the CheckV (37), with thresholds set at 95% ANI and 85% AF (options “-min_ani 95, -min_tcov 85, -min_qcov 0”) (55).

Finally, for infant data sets, we obtained 4,705 non-redundant species-level vOTUs in bulk and 5,736 vOTUs in VLP. And for adult data sets, we obtained 19,900 vOTUs in bulk and 24,389 vOTUs in VLP.

### Calculation of the abundance of vOTUs in each sample

To calculate the relative abundances of the different vOTUs in each sample, we performed two methods for building viral genome libraries using BWA v0.7.17-r1188 (39). We aligned the reads from each sample to a reference genome database to determine the mappings. We then calculated the reads per kilobase million (RPKM) value of vOTUs in each sample with CoverM v0.6.1 (https://github.com/wwood/CoverM) (The option is “--mapper bwa-mem, --min-read aligned-percent 95, --min-read-percent-identity 90, --min-covered fraction 75, --methods rpkm”). The “presence” of a vOTU was defined in a sample if over 75% of its length was covered by the aligned reads from the sample. vOTUs were not present in a sample (see the definition of the “presence” of a vOTU in a sample) and were excluded and set their relative abundances to zero. Relative species abundance was calculated by dividing the RPKM of a specific viral population by the total RPKM of all viral populations.

### Identification of completeness of vOTUs

We employed CheckV (37) v0.7.0 to assess the completeness of these genomes. A complete genome is defined as achieving a perfect score of 100%. Genomes with a completeness score above 90% will be classified as high quality, while those ranging from 50% to 90% will be considered medium quality. Genomes scoring below 50% will be categorized as low quality, and if no completeness estimate is available, they will be labeled as not determined. By utilizing CheckV, we can evaluate the completeness of these genomes and assign them to their respective quality categories. We performed a sequence comparison of the genomes obtained by the two methods using FastANI (56) (v1.1 --fragLen 100 --minFraction 0.85).

### Taxonomic annotation and host analysis

To provide accuracy and annotation rates for virus taxonomic classification, three distinct annotation tools were employed, and all of them are based on the International Committee on Taxonomy of Viruses (ICTV) taxonomy rules. VirusTaxo (57) compares the nucleotide sequences against its prebuilt database and has high accuracy at the genera of viruses. Usually, we classify the contigs with Entropy >0.5 as “unclassified,” and the rest as the actual prediction of the VirusTaxo. For those viruses not annotated by VirusTaxo, PhageGCN (58) was used. Finally, for any remaining unannotated viruses, we subjected them to geNomad (59) for taxonomic classification. The three software outputs were integrated as the annotation results. Based on the host information from the ICTV, we got the part that belongs to the eukaryotic virus. We excluded this part and used the remaining vOTUs to predict the lifestyle of phages as “Lytic” and “Temperate” using PHACTS (60) with default settings. Ten replicate PHACTS predictions were performed. Viral hosts were categorized based on their taxonomy using the RefSeq Genome database of NCBI.

### Bioinformatic analyses and statistics

All statistical analyses were performed in the R v4.2.3 platform. Alpha and beta diversity were assessed based on the relative abundance profile of bulk and VLP using the R package vegan. Data visualization was carried out using the ggplot2 package. The Spearman’s correlation coefficient was calculated using the cor. test function. Significance tests were performed using the function paired Wilcox. test with default parameters.

## Data Availability

These data were derived from the following resources available in the public domain: NCBI (https://www.ncbi.nlm.nih.gov/sra), and CNCB (https://www.cncb.ac.cn/). For adults, the public sequencing data are available in the CNCB GSA database (PRJCA007087) and the NCBI SRA database (PRJNA835720). For infants, the data can be found in the NCBI SRA database under accession PRJNA524703. All scripts underlying data analyses and Figures can be found at: https://github.com/yuyunmuzi/Bulk-VLP-Gut-Virome-infants-and-adults

## SUPPLEMENTAL MATERIAL

Supplemental figures spectrum.00013-25-s0001.docx Figures S1 to S10.

Table S1 spectrum.00013-25-s0002.xlsx details of the respective vOTUs in the bulk and VLP in the infant and adult datasets.

Table S2 spectrum.00013-25-s0003.xlsx LDA effect size of genera in bulk and VLP in the infant and adult datasets.

ASM does not own the copyrights to Supplemental Material that may be linked to, or accessed through, an article. The authors have granted ASM a non-exclusive, world-wide license to publish the Supplemental Material files. Please contact the corresponding author directly for reuse.
