# Supplementary material for: Biases and complementarity in gut viromes obtained from bulk and virus-like particle-enriched metagenomic sequencing
Source: Microbiol Spectr. 2025 Jul 2;13(8):e00013-25. doi: 10.1128/spectrum.00013-25 (PMC12323601; doi:10.1128/spectrum.00013-25)
Supplement: Supplemental figures — Figures S1 to S10. [file spectrum.00013-25-s0001.docx]

## Supplementary material

Supplementary Figures:


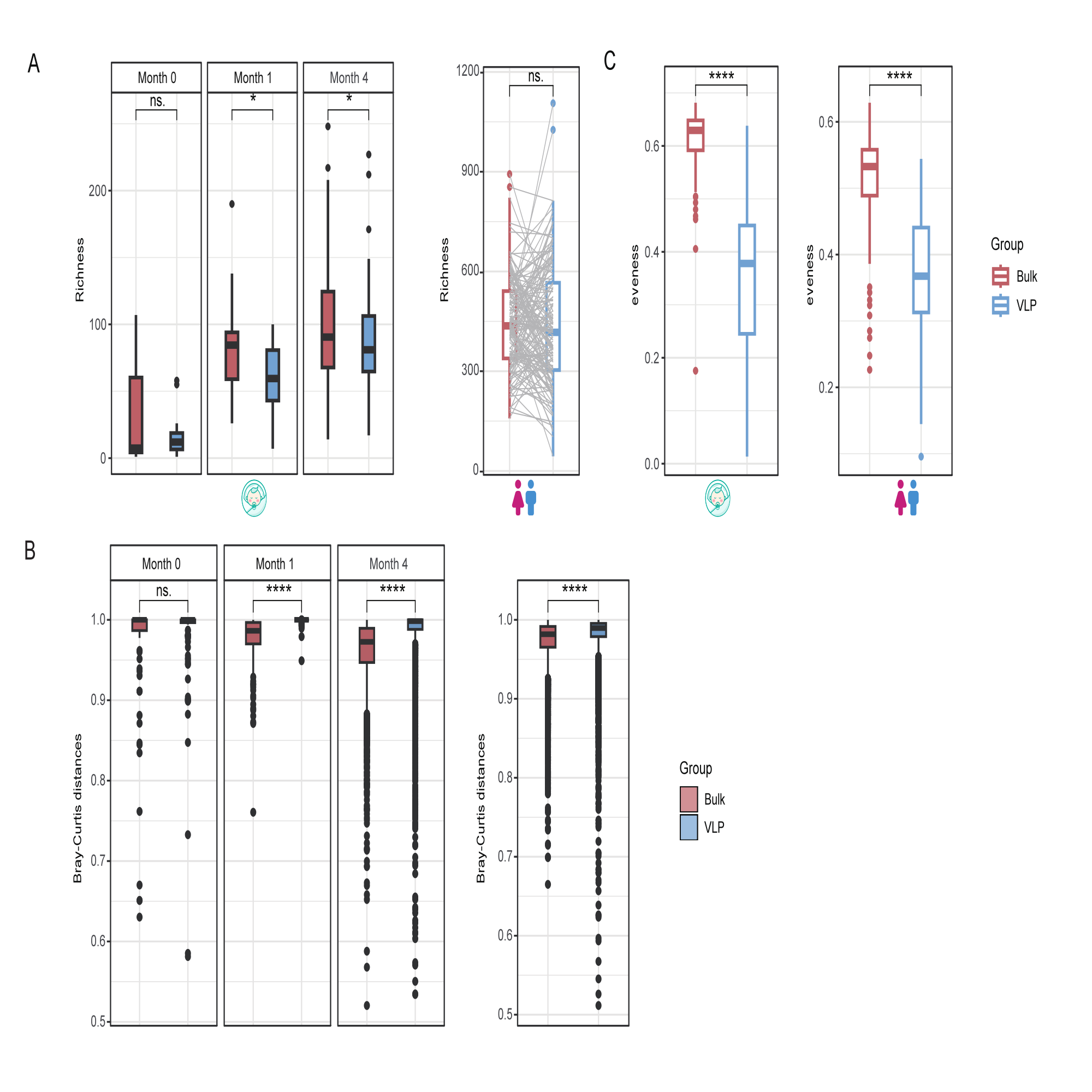


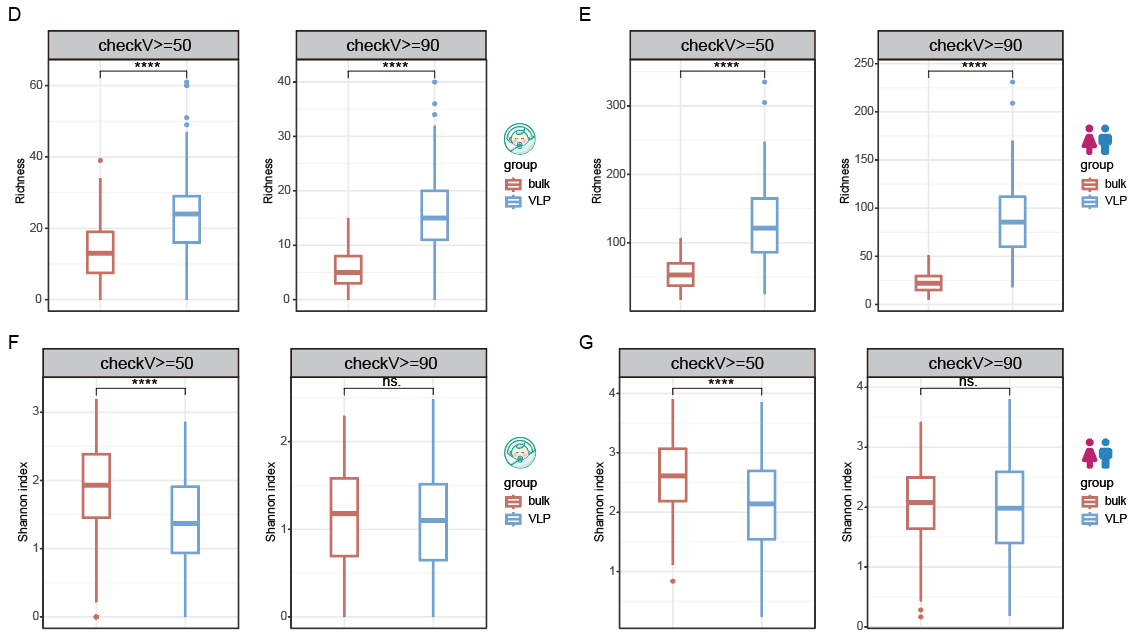


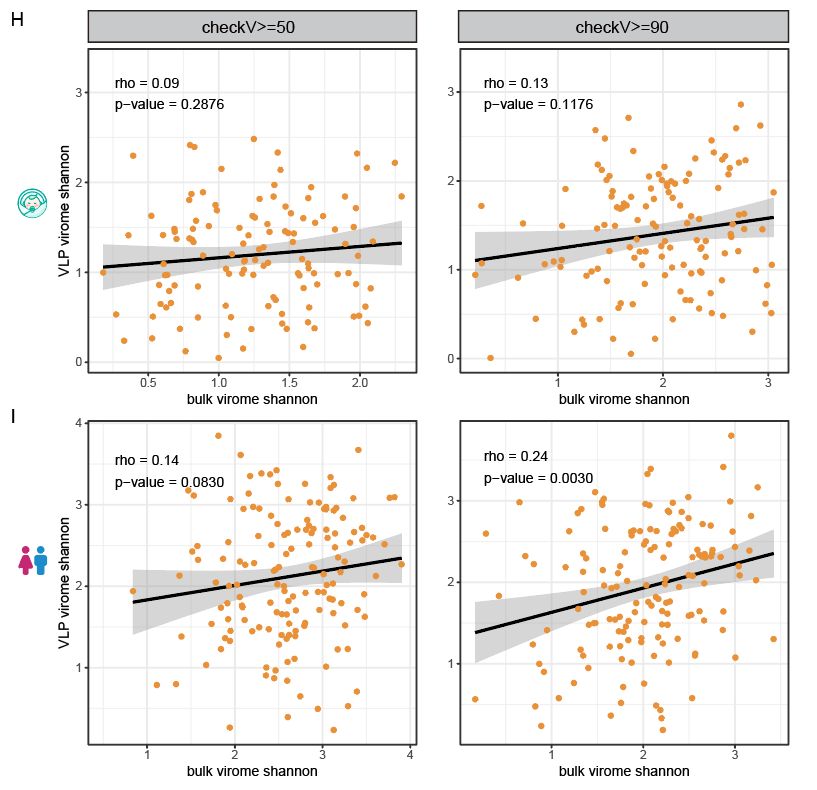


**Supplementary Figure S1**: The different characteristics of alpha diversity and beta diversity of vOTUs in bulk- and viral-like particle- (VLP) metagenome sequencing samples. A, Comparison of within-sample observed vOTUs (richness, Left for infants, and right for adults). B, Comparison of within-sample evenness of vOTUs (Left for infants, and right for adults). C, Comparison of between-sample dissimilarities observed vOTUs (Bray-Curtis distances, Left for infants, and right for adults). D-E, Comparison of within-sample observed vOTUs (richness, Left for CheckV >=50%, and right for CheckV >=90%). D is for infants, E is for adults. F-G, Comparison of within-sample Shannon diversity (Left for CheckV >=50%, and right for CheckV >=90%). F is for infants, G is for adults. H-I, Comparison of within-sample Shannon diversity of vOTUs (Left for CheckV >=50%, and right for CheckV >=90%). H is infants, I is adult. Paired Wilcoxon rank-sum test: ns.: p>0.05; *p< 0.05; **p <0.01, ***p<0.001, ****p<0.0001. Colors refer to groups: bulk (red) and VLP (blue).


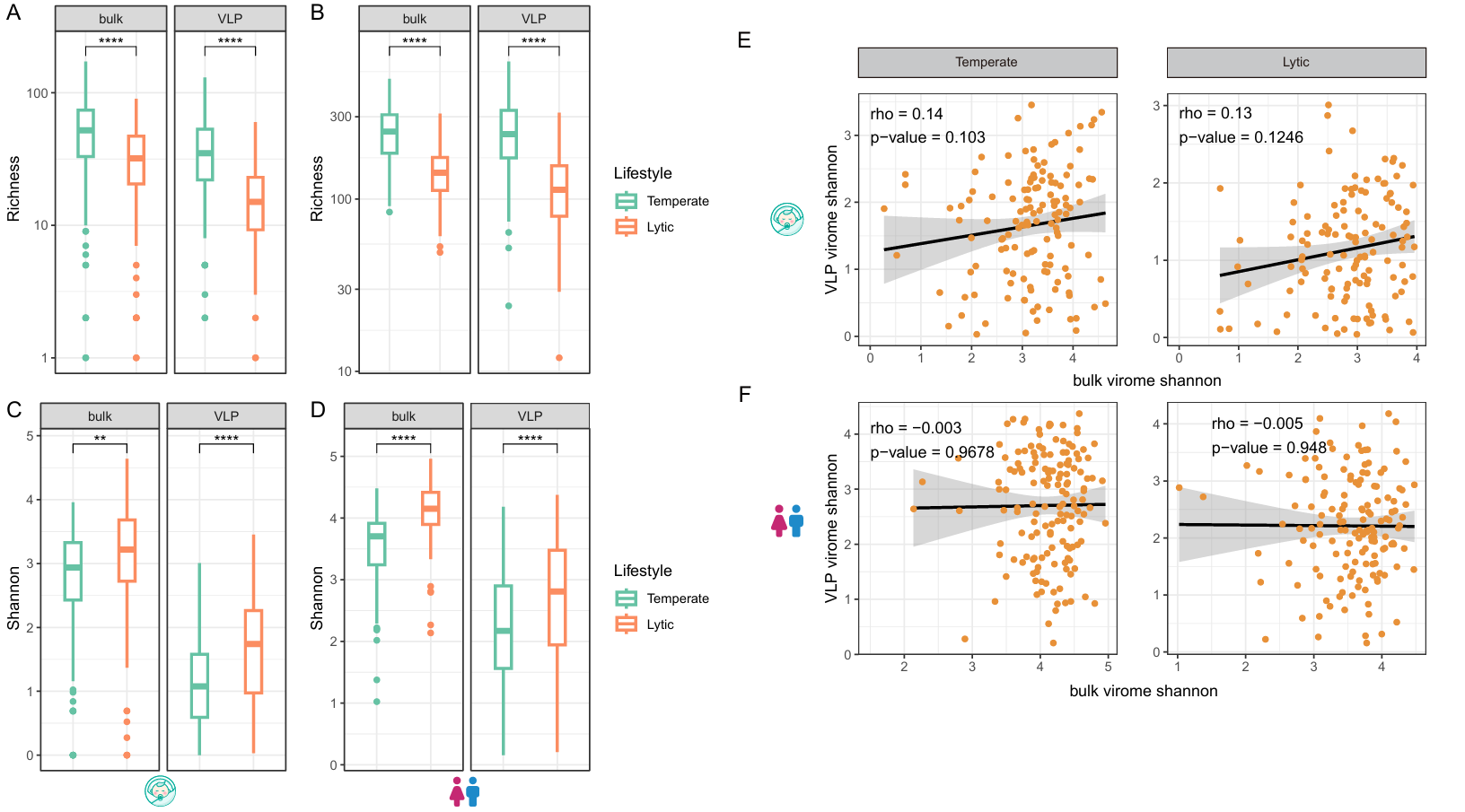


**Supplementary Figure S2**: The different characteristics of alpha diversity of temperate and lytic

vOTUs in bulk- and viral-like particle- (VLP) metagenome sequencing samples. A-B, Comparison of richness of temperate and lytic vOTUs in bulk and VLP samples (Left for infants, and right for adults). C-D, Comparison of within-sample Shannon diversity of temperate and lytic vOTUs in bulk and VLP samples (Left for infants, and right for adults). Wilcoxon rank-sum test: ns.: p>0.05; *p < 0.05; **p < 0.01, ***p<0.001, ****p<0.0001. Colors refer to groups: Temperate (green) and Lytic (orange). E-F, Spearman’s rank correlation of alpha diversity (Shannon index, “Temperate” of the virome on the left and the “Lytic” of the virome on the right) between bulk-virome and VLP-virome with two sides. E is infants, and F is adults.


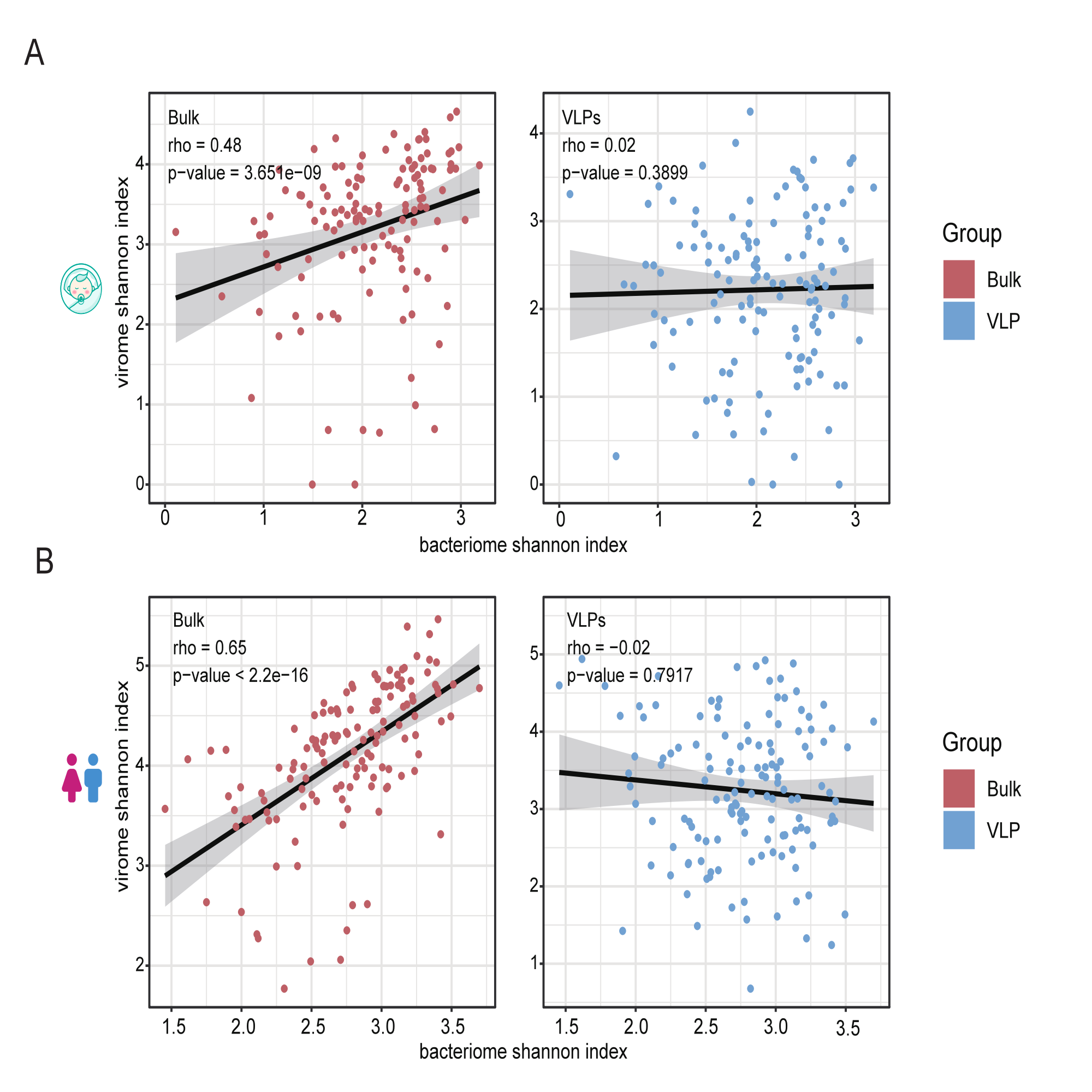


**Supplementary Figure S3**: Spearman’s rank correlation of alpha diversity (Shannon index) between virome and bacteriome with two sides. A is infants, and B is adults (Left is for bulk, and right is for VLP).


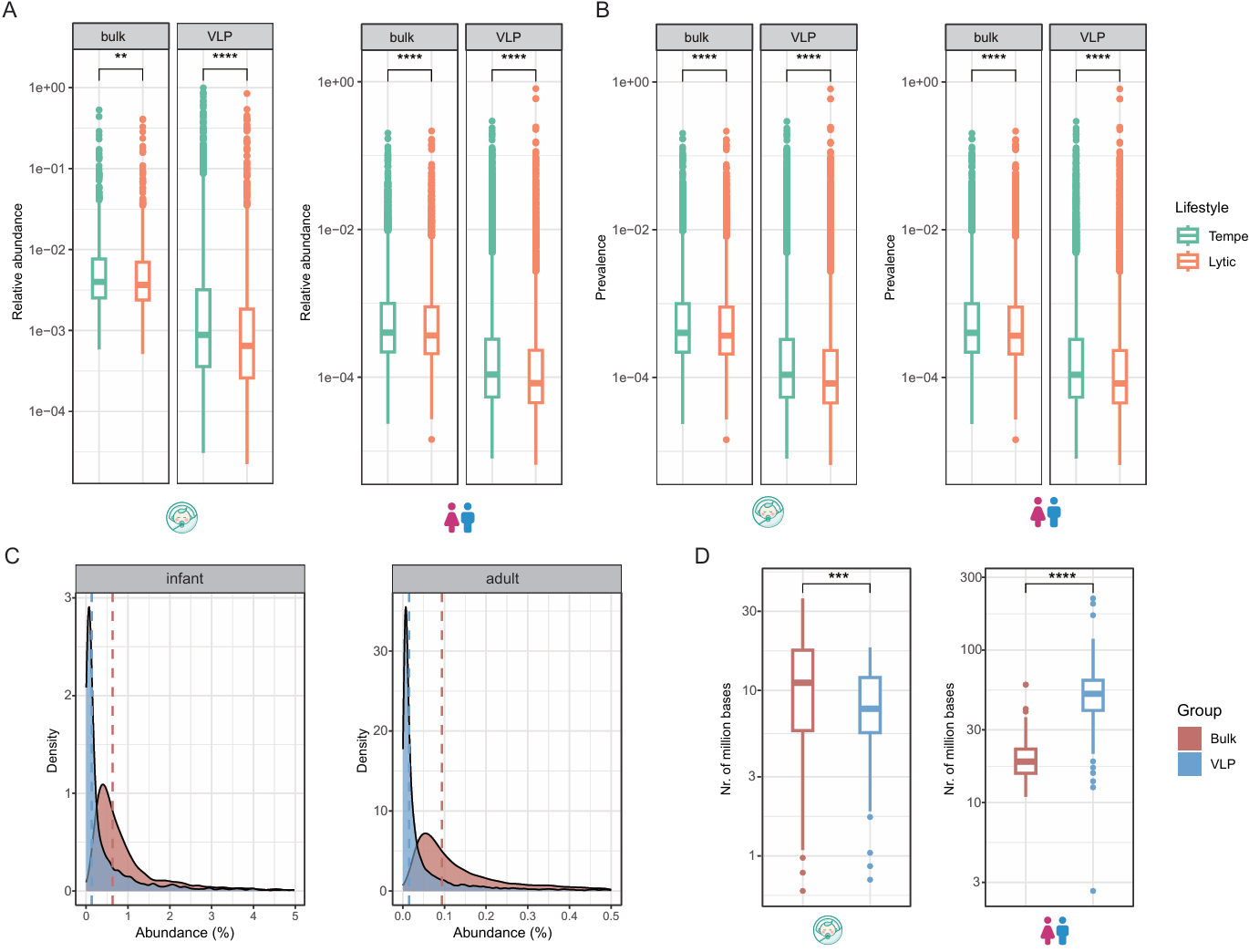


**Supplementary Figure S4**: The distribution of the relative abundance of vOTUs and the sequencing depth in bulk- and viral-like particle- (VLP) metagenome sequencing samples. A, The comparison of the relative abundance of temperate and lytic vOTUs in bulk and VLP samples (Left for infants, and right for adults). B, The comparison of the prevalence of temperate and lytic vOTUs in bulk and VLP samples (Left for infants, and right for adults). Wilcoxon rank-sum test: ns.: p>0.05; *p < 0.05; **p < 0.01, ***p<0.001, ****p<0.0001. Colors refer to groups: Temperate (green) and Lytic (orange). C, The distribution of the relative abundance of vOTUs (overlap) (Left for infants and the relative abundance <5%, right for adults and the relative abundance <0.5%). D, The sequencing depth of gut VLPs enriched and bulk metagenomes distributed (Left for infants, and right for adults). Paired Wilcoxon rank-sum test: ns.: p>0.05; *p < 0.05; ** p < 0.01, ***p<0.001, ****p<0.0001. Colors refer to groups: bulk (red) and VLP (blue).


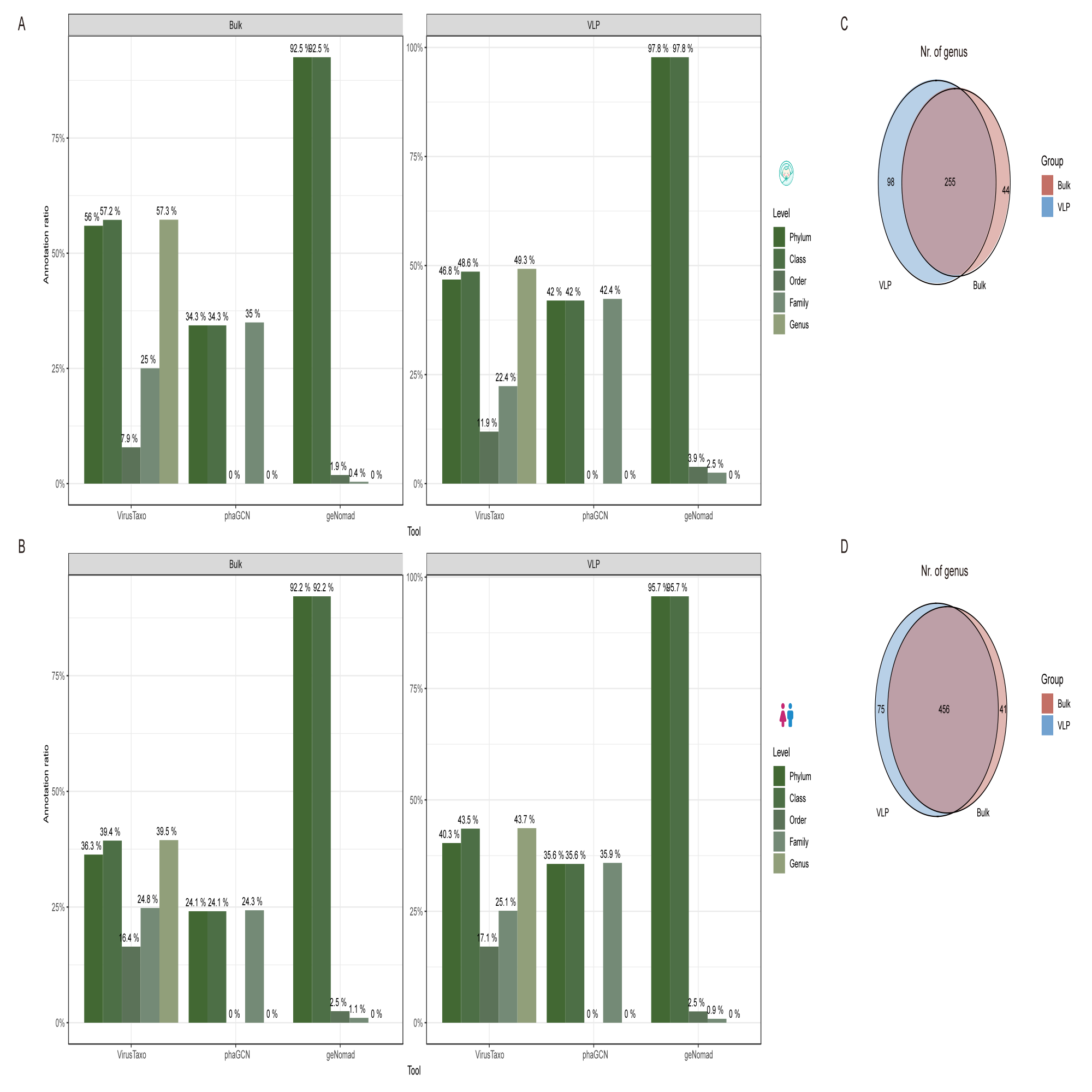


**Supplementary Figure S5**: A-B, the annotation ratio of different taxonomic tools at different levels (From Phylum to Genus). C-D, Veen plot shows the overlap and uniqueness of the annotation in the genus of vOTUs by two methods. Colors refer to groups: bulk (red) and VLP (blue).


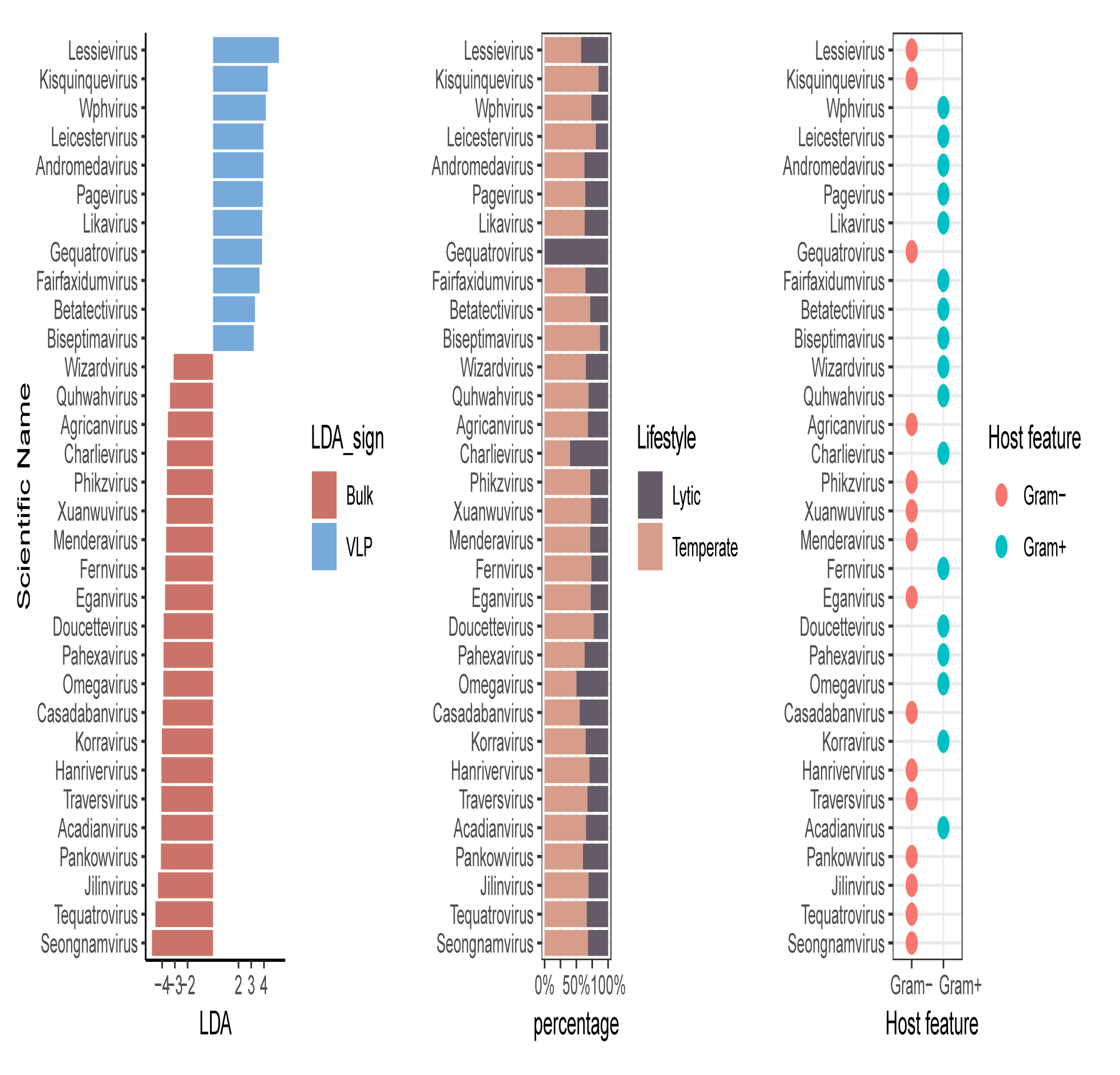


**Supplementary Figure S6**: Enrichment of genera in bulk and VLP in infants. The left panel shows that 32 genera are unique in infant datasets (11 were enriched in VLP and 21 were enriched in bulk). The middle panel illustrates the lifestyle characteristics of these genera, and the right panel depicts the host features associated with these genera.


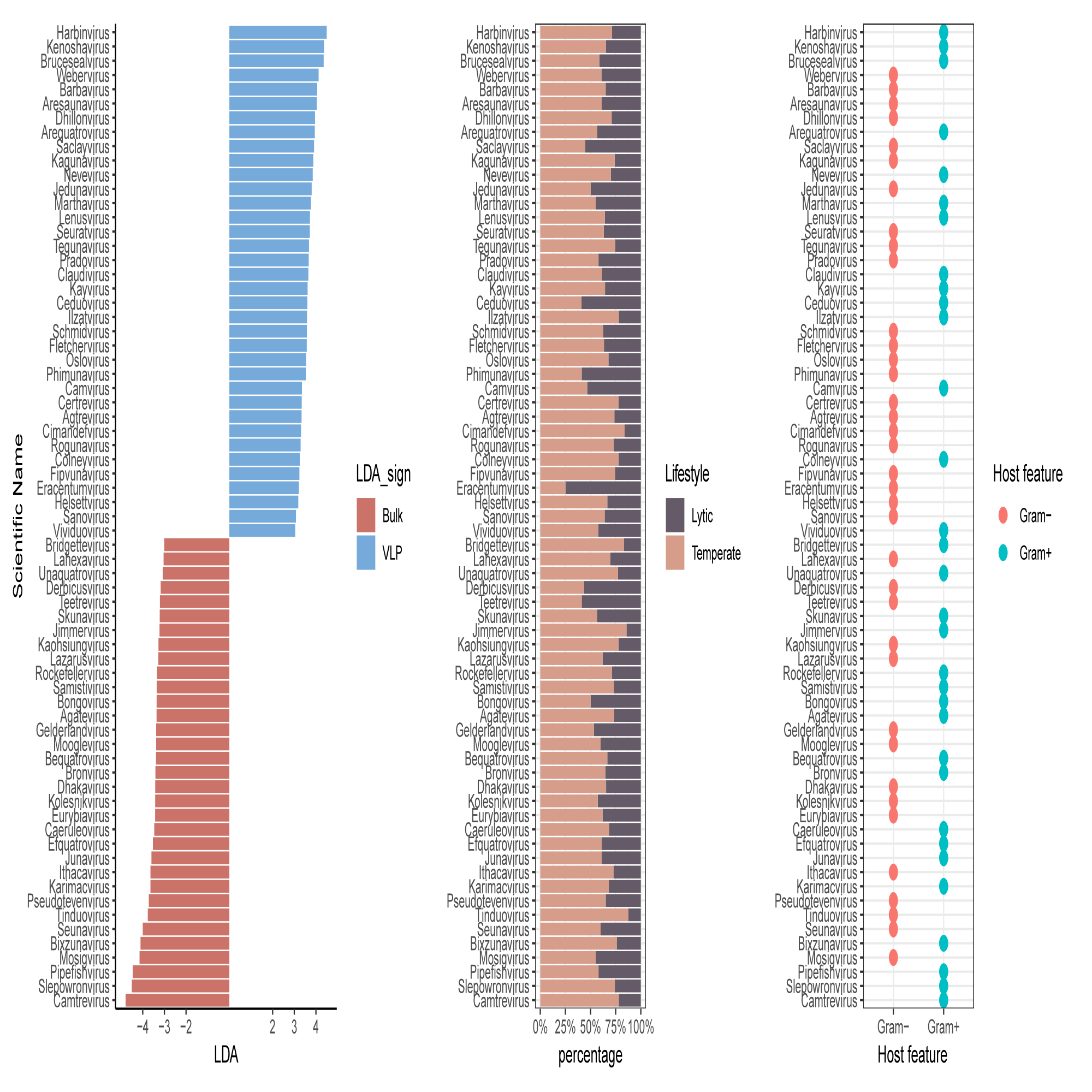


**Supplementary Figure S7**: Enrichment of genera in bulk and VLP in adults. The left panel shows that 65 genera are unique in adult datasets (32 were enriched in VLP and 33 were enriched in bulk). The middle panel illustrates the lifestyle characteristics of these genera, and the right panel depicts the host features associated with these genera.


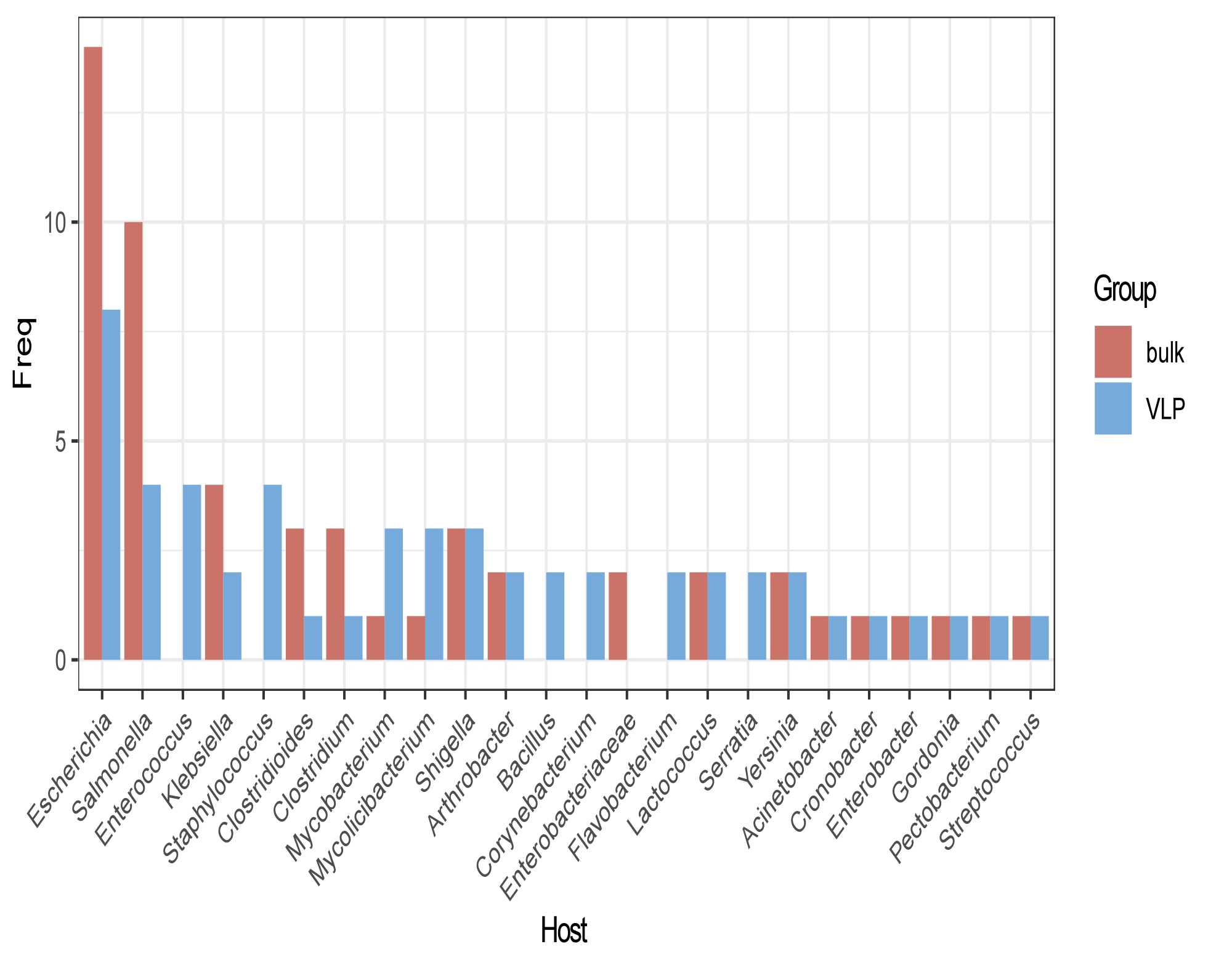


**Supplementary Figure S8**: Host distribution of phage genera co-occurring in the adult and infant.


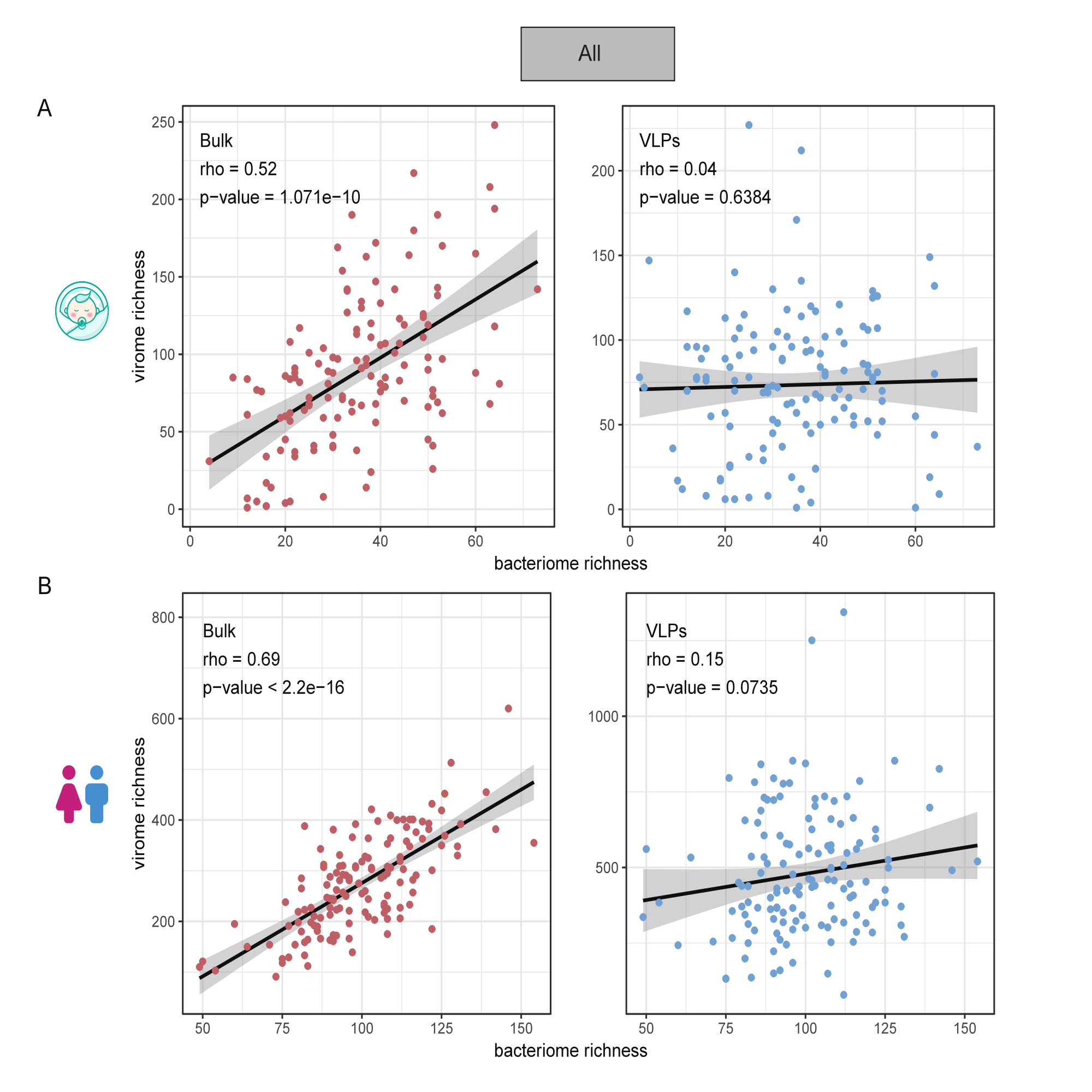


**Supplementary Figure S9**: Spearman’s rank correlation of alpha diversity (richness) between virome and bacteriome with two sides. A is infants, and B is adults (Left is for bulk, and right is for VLP).


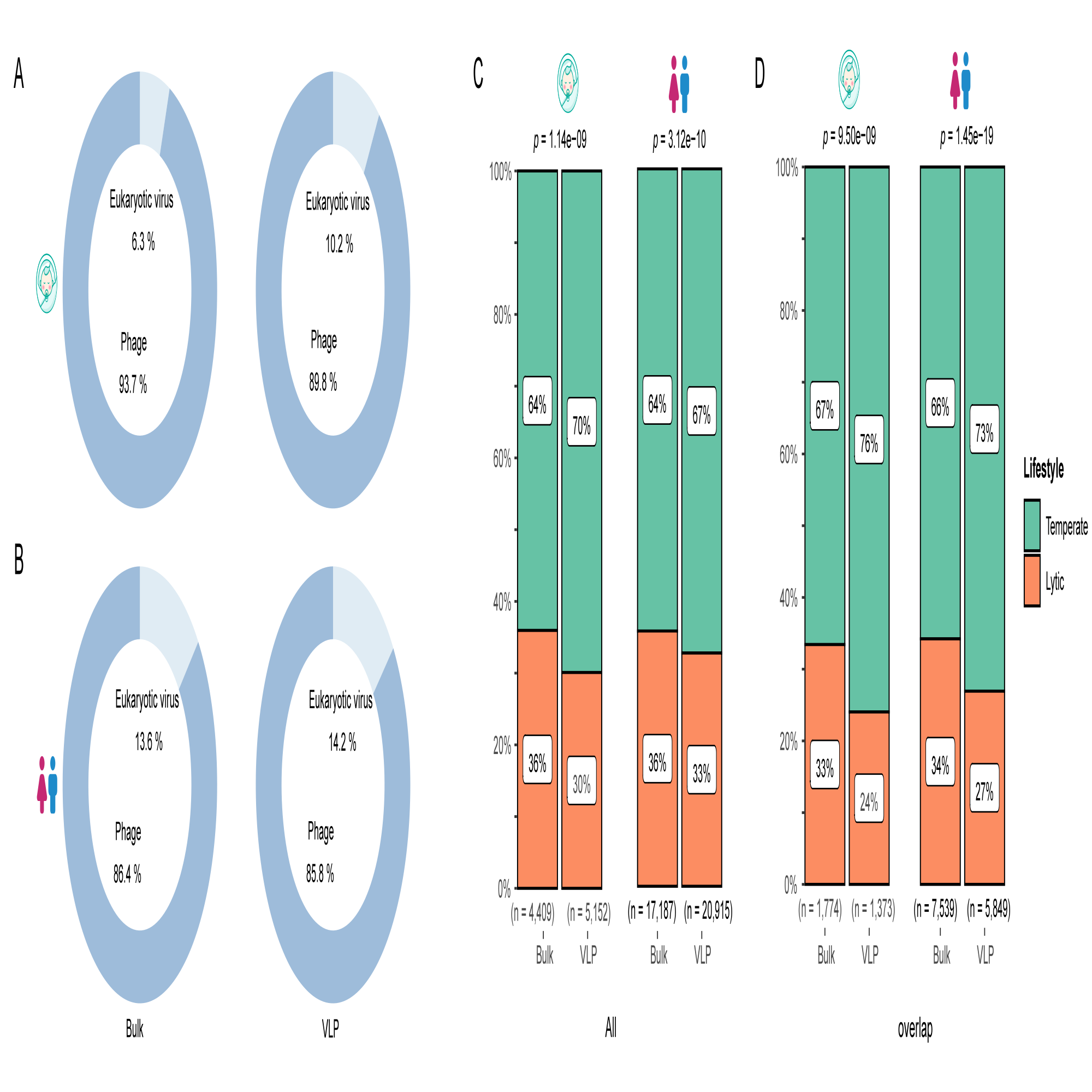


**Supplementary Figure S10**: A-B, The portion of eukaryotic viruses in Bulk and VLP in the infant and adult datasets. C-D, Distribution of phage lifestyle in Bulk and VLP in the infant and adult datasets. C is all, D is overlap.

Supplementary Tables:

**Supplementary Table S1**: The details of the respective vOTUs in the bulk and VLP in the infant and adult datasets.

**Supplementary Table S2**: The LDA EFfect Size of genera in bulk and VLP in the infant and adult datasets.
